# Three-in-one approach towards efficient organic dye-sensitized solar cells: aggregation suppression, panchromatic absorption and resonance energy transfer

**DOI:** 10.3762/bjnano.8.171

**Published:** 2017-08-17

**Authors:** Jayita Patwari, Samim Sardar, Bo Liu, Peter Lemmens, Samir Kumar Pal

**Affiliations:** 1 Department of Chemical, Biological and Macromolecular Sciences, S. N. Bose National Centre for Basic Sciences, Block JD, Sector III, Salt Lake, Kolkata 700 106, India; 2 Institute for Condensed Matter Physics, TU Braunschweig, Mendelssohnstraße 3, 38106 Braunschweig, Germany; 3 Laboratory for Emerging Nanometrology, TU Braunschweig, Braunschweig, Germany

**Keywords:** anti-aggregation, co-sensitization, dye-sensitized solar cells (DSSC), Förster resonance energy transfer (FRET), NIR harvesting, panchromatic absorption

## Abstract

In the present study, protoporphyrin IX (PPIX) and squarine (SQ2) have been used in a co-sensitized dye-sensitized solar cell (DSSC) to apply their high absorption coefficients in the visible and NIR region of the solar spectrum and to probe the possibility of Förster resonance energy transfer (FRET) between the two dyes. FRET from the donor PPIX to acceptor SQ2 was observed from detailed investigation of the excited-state photophysics of the dye mixture, using time-resolved fluorescence decay measurements. The electron transfer time scales from the dyes to TiO_2_ have also been characterized for each dye. The current–voltage (*I*–*V*) characteristics and the wavelength-dependent photocurrent measurements of the co-sensitized DSSCs reveal that FRET between the two dyes increase the photocurrent as well as the efficiency of the device. From the absorption spectra of the co-sensitized photoanodes, PPIX was observed to be efficiently acting as a co-adsorbent and to reduce the dye aggregation problem of SQ2. It has further been proven by a comparison of the device performance with a chenodeoxycholic acid (CDCA) added to a SQ2-sensitized DSSC. Apart from increasing the absorption window, the FRET-induced enhanced photocurrent and the anti-aggregating behavior of PPIX towards SQ2 are crucial points that improve the performance of the co-sensitized DSSC.

## Introduction

The increasing demand for fossil-fuel energy sources and the intensifying environmental pollution have promoted an extensive research for the development of efficient conversion technologies of clean and renewable energy sources. Solar energy has been considered to be the most promising sustainable and renewable energy source because of its quasi-unlimited supply. In the last decade, dye-sensitized solar cells (DSSCs) have drawn significant attention as an alternative conversion technology for solar energy, besides conventional Si-based solar cells, because of its simple and less expensive processing and a wide range of potential applications [1–3]. Since the first invention of DSSCs by O’Regan and Grätzel in 1991 [4], a plethora of designing strategies has been developed and reported in the literature in order to optimize the device performance and to minimize the effective costs [5–10].

A good spectral match between the absorption spectra of the sensitizer and the incident solar radiation is an essential requirement for efficient conversion of solar energy [11]. So far, the most widely used photosensitizers are ruthenium (Ru)-based dyes (e.g., N719, N3, Black dye) because of their good stability and efficient performance. However, the scarcity of the raw material, the enormous purification cost, the low molar extinction coefficient (ε) of the metal-to-ligand charge transfer (MLCT) band, a poor absorption in the near-infrared (NIR) range of the solar light and the toxicity are well-documented limitations of these Ru photosensitizers [12]. As an alternative to Ru dyes, less toxic and less expensive organic dyes are being used to sensitize the photoanodes [13]. Organic dyes are attractive as a photosensitizer because of their high molar extinction coefficient, tunable absorption wavelength, and easy design and synthesis strategies. Still, the efficiency of organic dye-sensitized solar cells is not comparable to that of the metallo-organic dyes because of their narrow absorption spectra, shorter excited-state lifetimes and the problem of self-aggregation on the semiconductor surface [14]. For efficient organic dye-sensitized solar cells, appreciated techniques to achieve a broad absorption window are either using a sensitizer solution cocktail mixing one dye absorbing in the visible region with another dye absorbing in the NIR region or incorporating Förster resonance energy transfer (FRET) between two co-sensitizers [9,15–19]. However, co-sensitization brings some additional complexity to the effective performance of the device [20–21]. The control of the uptake of different dyes, the prevention of unwanted reactions and unfavorable electron–hole recombination [22] affect the device performance. FRET has been proposed to be a more useful tool to achieve strong light harvesting over a broad wavelength range without affecting the key parameters such as open circuit voltage (*V*_oc_) or fill factor (FF) of the DSSCs [23]. An enhancement in photocurrent due to efficient energy transfer from a quantum dot to the sensitizing dye has been reported earlier for quantum dot co-sensitized DSSCs [24–25]. But the study of energy transfer between two dyes in co-sensitized DSSCs is sparsely reported in the literature. Aggregation-induced self-quenching of excited-state electrons of NIR-absorbing dyes is another limitation widely reported in co-sensitized DSSCs [26–27]. The required use of high concentrations of co-adsorbent is an unavoidable constraint on the co-sensitization of organic dyes [18,28–30]. In order to avoid the co-adsorbent, one of the co-sensitizers can be used to counteract the aggregation of the other. Spectroscopic studies and excited-state dynamics of the two dyes can be helpful to optimize the co-sensitization procedure and to understand the complicated set of electron and energy transfer processes occurring in co-sensitized DSSCs.

We have chosen squarine (SQ2) and protoporphyrin IX (PPIX) to fabricate a co-sensitized DSSC and detailed photo-physical studies have been carried out to explore the dynamical processes occurring between these two dyes after the attachment to the TiO_2_ surface. PPIX is an environmentally friendly organic sensitizer that has a strong absorption in the visible region of the solar spectrum. Application of PPIX as a sensitizer in DSSCs has been reported in our earlier publications [31]. SQ2 is a commercially available NIR-absorbing dye that has been receiving growing attention in the field of DSSCs due to its light-harvesting ability in the NIR region [32–33]. From the spectral overlap between the absorption of SQ2 and emission of PPIX and from the time-resolved fluorescence decay of the mixture of SQ2 and PPIX, FRET from PPIX to SQ2 has been confirmed. The DSSCs were co-sensitized with different molar ratios of the two dyes. The FRET-enhanced photocurrent and the anti-aggregating properties of PPIX have been experimentally proven to be the major reasons for the enhanced light-harvesting over the entire solar spectrum. PPIX–SQ2 co-sensitized DSSCs were found to be more efficient compared to SQ2-sensitized DSSCs prepared using the optimum concentration of the well-known co-adsorbent chenodeoxycholic acid (CDCA). The wavelength-dependent photocurrent measurements reveal that a small amount of PPIX can efficiently act as a co-adsorbent instead of CDCA. CDCA is more costly than PPIX, and the required amount of CDCA to suppress aggregation is also much higher than PPIX. Thus, addition of PPIX as a co-sensitizer to SQ2-sensitized DSSC provides an increased absorption window, FRET-enhanced photocurrent and the prevention of SQ2 aggregation.

## Experimental

### Reagents

TiO_2_ nanoparticles (21 nm), protoporphyrin IX (PPIX), platinum chloride (H_2_PtCl_6_), lithium iodide (LiI), 4-*tert*-butylpyridine (TBP) and iodine (I_2_) were purchased from Sigma-Aldrich. Squarine (SQ2) and 60 μm thick Surlyn were bought from Solaronix. Dimethyl sulfoxide (DMSO), acetonitrile and ethanol (≥99%) were purchased from Merck, and Ultrapure water was obtained from Millipore System, (18.2 MΩ·cm). The conducting glass substrate with fluorine-doped tin oxide (FTO) was purchased from Sigma-Aldrich. FTOs were cleaned by successive sonication with soap water, acetone, ethanol and deionized (DI) water for 10 min each and were then dried prior to their usage.

### Optical characterization

A Shimadzu UV-2600 spectrophotometer and a Jobin Yvon Fluoromax-3 fluorimeter have been used for the collection of the steady-state absorption spectra and the emission spectra, respectively. The solid-state absorption spectra were recorded in reflecting mode using a STS-VIS-L10-400-SMA spectrograph with wavelength resolution of 0.47 nm. For the transmission and collection of light, a lab-grade optical fiber probe from Ocean Optics was used in this setup. The picosecond time-resolved spectroscopic studies have been carried out using a commercial time-correlated single-photon counting (TCSPC) setup from Edinburgh Instruments. Picosecond pulsed lasers of 633 nm and 409 nm wavelengths have been used as excitation sources in this study. The instrument response function was 80 ps. The experimental setup and methodology were discussed in detail in our earlier publications [34–35]. FRET between the donor (PPIX) and the acceptor (SQ2) was studied using traditional methodology [36] by calculating the Förster distance (*R*_0_ in Å). The details of the calculation procedure are mentioned in the earlier publications of our group [37–38].

### Assembly of DSSCs

TiO_2_-coated FTO glass substrates were annealed at 450 °C for 1 h, followed by a cooling to 80 °C and immersing into the dye solution for 24 h. We used six different dye cocktails as sensitizers in this study, prepared by mixing different molar ratios of the two dyes, SQ2 and PPIX. Initially a 0.3 mM solution of SQ2 was prepared in ethanol and a PPIX solution of the same concentration was prepared using DMSO as a solvent. These two solutions were mixed in different ratios maintaining a total volume of 10 mL. The ratios of SQ2/PPIX used are 10:0, 8:2, 6:4, 4:6, 2:8 and 0:10. For the one-by-one sensitization technique, the photoanodes were immersed in a single-dye solution for 12 h, followed by 12 h immersion in the solution of the other dye. The one-by-one sensitized DSSCs are named SQ2_PPIX and PPIX_SQ2 according to the sequence of dye used for sensitization. To ease the comparison, the total immersion time was 24 h during both mixed-dye sensitization and sequential sensitization. The counter electrodes were prepared by depositing platinum on the FTO substrates via thermal decomposition of 5 mM platinum chloride (in isopropanol) at 385 °C for 30 min. 60 μm thick Surlyn was used as a spacer between the active and counter electrodes. The I^−^/I_3_^−^ redox couple, which was prepared by mixing iodine crystal (I_2_), lithium iodide (LiI), and 4-*tert*-butylpyridine (TBP) using acetonitrile as a solvent, was used as electrolyte. The active area of all the devices were 0.64 cm^2^.

### Device characterization

A Keithley multimeter was used to record the photocurrent–voltage (*I*–*V*) characteristics of the DSSCs, under 1 sun (100 mW cm^−2^) irradiance (AM 1.5 simulated illuminations, Photo Emission Tech). The equations used to calculate the fill factor (FF) and power conversion efficiency (η) of the solar cells are

[1]
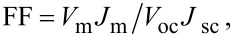


[2]
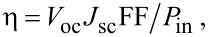


where *V*_m_ and *J*_m_ are the voltage and current density at the maximum power output, respectively, *J*_sc_ is the short-circuit photocurrent density and *V*_oc_ is the open-circuit photovoltage of the solar cell. The intensity of the incident light (*P*_in_) is 100 mW·cm^−2^. The wavelength-dependent photocurrent was measured using a custom-made setup, which consists of a Bentham monochromator and two light sources (tungsten and xenon).

## Results and Discussion

**Figure 1:**
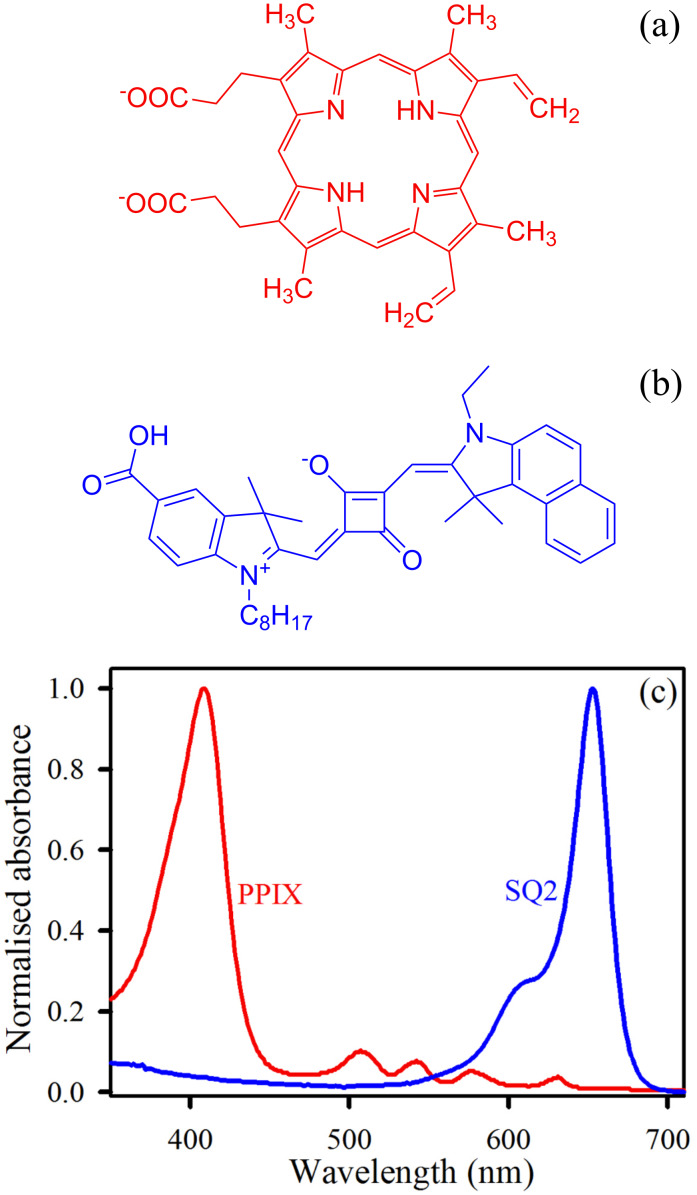
Structure of (a) PPIX and (b) SQ2; (c) normalized absorption spectra of PPIX in DMSO and SQ2 in ethanol.

In the present study, the two organic dyes chosen for co-sensitization are protoporphyrin IX (PPIX) and squarine (SQ2) because of their complementary absorption spectra. From the structure of PPIX (Figure 1a), it is evident that this sensitizer is a derivative of porphyrin, which is an integral part of many naturally occurring and biologically active macrocyclic compounds, e.g. hemoglobin and chlorophyll. The absorption of PPIX in DMSO is shown in Figure 1c, where the highest intensity peak appearing at 407 nm is due to the Soret band and the other four lower intensity peaks at 509, 542, 578 and 630 nm are the Q-bands. The use of PPIX as a green alternative of the conventional toxic and expensive sensitizers has been reported earlier [31], but the so far reported efficiencies of PPIX-sensitized DSSCs are poor from a commercialization point of view. In spite of having a high molar extinction coefficient in the visible region, PPIX shows a very low efficiency probably because of the inadequate absorbance in the NIR region. Squarines are widely known red/NIR-absorbing organic dyes that can be used as a co-sensitizer with a visible absorbing dye to get a wide panchromatic absorption. From the structure of SQ2 (Figure 1b) and the absorption spectra of SQ2 in ethanol (Figure 1c), it can be correlated that the highest intensity peak appearing at 651 nm corresponds to π–π* charge-transfer (CT) transitions. A lower intensity peak at the blue end (604 nm) of the spectra is a notable signature of dye aggregation [27]. It is evident from the combined absorption spectra shown in Figure 1c that by using PPIX and SQ2 as co-sensitizers a wide range of the solar spectrum, from 360 nm to 680 nm, can be harvested.

**Figure 2:**
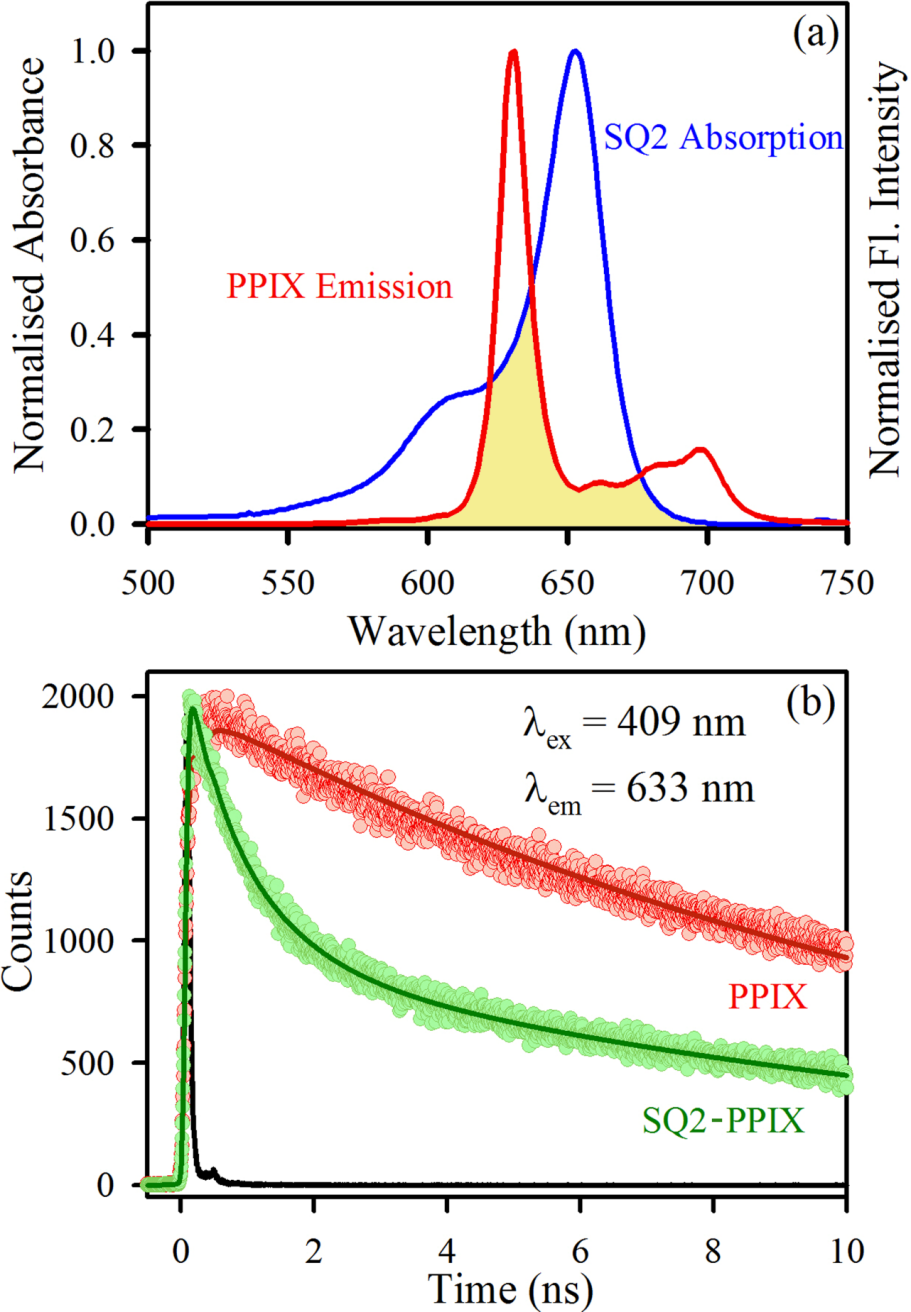
(a) Spectral overlap between the emission of PPIX and the absorption of SQ2, (b) The picosecond-resolved fluorescence decays of PPIX attached to Al_2_O_3_ in absence (red) and presence (green) of SQ2.

Before going into the details of the co-sensitized DSSCs using PPIX and SQ2, the probable complexities and advantages of these dye mixtures were studied using ultrafast spectroscopic studies. A significant overlap between the absorption of SQ2 and the emission of PPIX was observed (Figure 2a). So, there is a possibility of energy transfer from the excited state of PPIX to SQ2 when the two dyes come to close proximity. To create this proximity between these two dyes Al_2_O_3_ has been used. The excited-state lifetime of PPIX (attached to Al_2_O_3_) was measured in presence and absence of SQ2 by fitting the time-resolved fluorescence decays (Figure 2b). It can be noted from the lifetime components summarized in Table 1 that the average lifetime of the PPIX excited state (attached to Al_2_O_3_) is shortened from 13.20 to 5.47 ns when SQ2 is added to the solution. We propose FRET between the donor (PPIX) and the acceptor (SQ2) as the mechanistic explanation of the shortened lifetime of the excited donor state. The calculated distance between the donor and acceptor was found to be 4.9 nm with a 58% energy transfer efficiency. The confirmation of FRET from PPIX to SQ2 increases the possibility of getting enhanced photon-to-current conversion in the visible region. Again because of the long lifetime of the excited state of PPIX, both electron transfer to TiO_2_ and energy transfer to SQ2 become feasible. Although these two processes will be competing with each other, the FRET from PPIX to SQ2 was observed to be efficient in terms of device performance and cost effectiveness, as discussed in the explanation of the device characteristics.

**Table 1:** Excited-state lifetime of the donor PPIX attached to Al_2_O_3_ in presence and absence of the acceptor SQ2.

sample	excitation wavelength (nm)	emission wavelength (nm)	τ_1_ (ns)	τ_2_ (ns)	τ_3_ (ns)	τ_avg_ (ns)

PPIX	409	633	13.20 (100%)	—	—	13.20
PPIX-SQ2	409	633	0.25 (23%)	1.02 (39%)	13.20 (38%)	5.47

**Figure 3:**
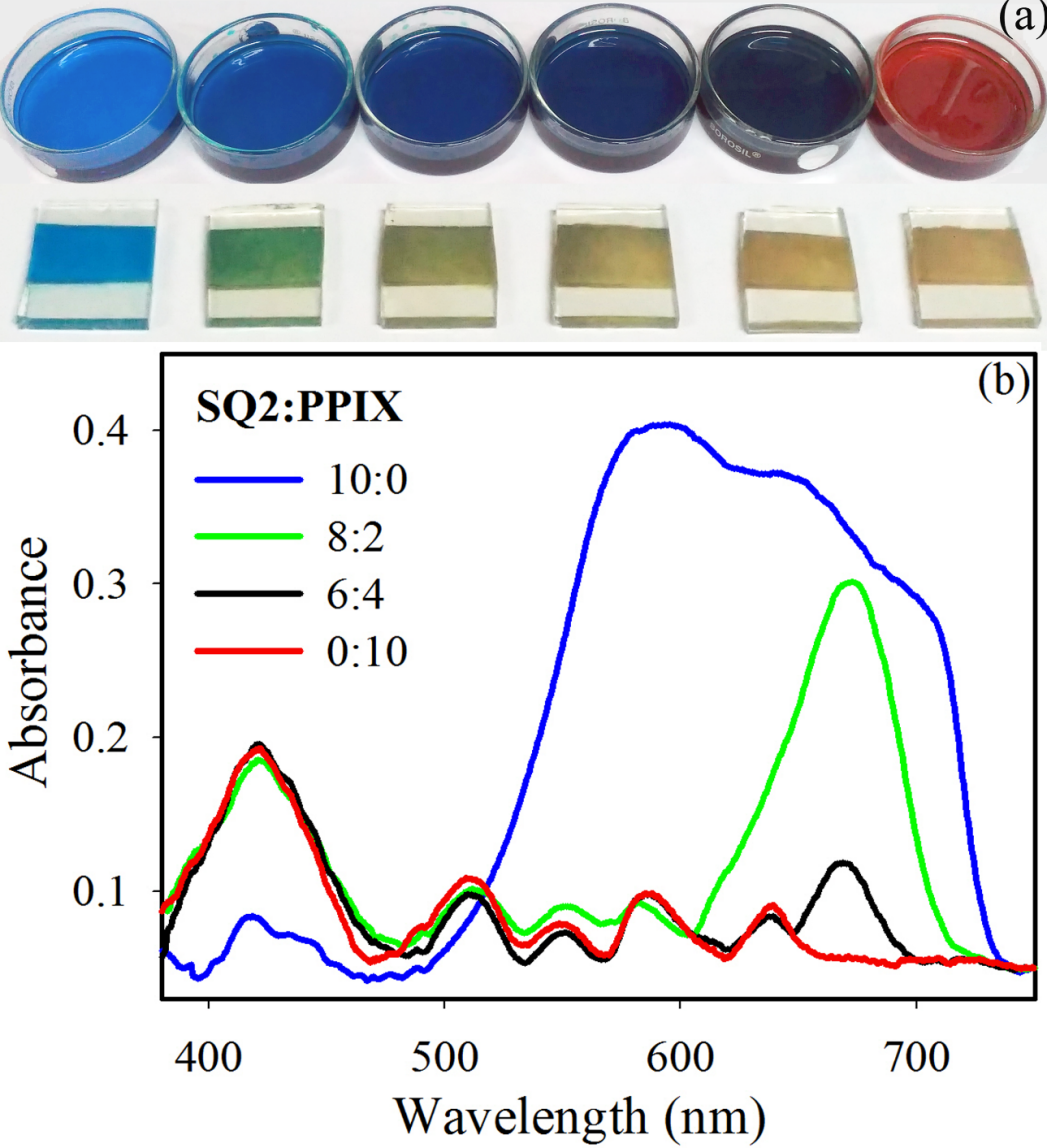
(a) Dye cocktail solutions and dye-sensitized TiO_2_ photoanodes with different molar ratios of SQ2 and PPIX. Starting from left to right the molar ratios of SQ2/PPIX are 10:0, 8:2, 6:4, 4:6, 2:8, 0:10. (b) Reflection-mode absorption spectra of TiO_2_ films sensitized with dye mixtures of different molar ratios of SQ2/PPIX.

The molar ratios of the two dyes SQ2/PPIX in the six cocktail mixtures used in this study were 10:0, 8:2, 6:4, 4:6, 2:8 and 0:10 and the sensitized DSSCs are named as S10P0, S8P2, S6P4, S4P6, S2P8 and S0P10, respectively. The different colors of the dye cocktails and the sensitized photoanodes are shown in Figure 3a. The solid-state absorption spectra of the selective photoanodes are shown in Figure 3b as a function of the dye loading. The scattering correction in the solid-state absorption spectra was done as reported in [39–40]. It is evident from the absorption spectra of the S10P0-sensitized photoanode that the absorption of SQ2 has broadened after attaching to a TiO_2_ surface compared to the liquid-phase absorption spectrum of SQ2 (Figure 1c). The broadening of the overall spectrum and increase in peak-intensity at lower wavelengths signify the increased aggregation after adsorption on a solid surface. The self-aggregation of squarine-based sensitizers is a familiar issue that decreases the efficiency of the device. In order to reduce the aggregation problem, the use of a co-adsorbent is a common approach described in literature. From the absorption spectra of the photoanodes S8P2 and S6P4, it can be observed that the simultaneous attachment of PPIX and SQ2 on the TiO_2_ surface can significantly reduce the aggregation of the SQ2 dye. It can be noted that after the addition of PPIX, the absorption region of SQ2 becomes narrower and the prominent peak around 600 nm, due the dimerization of SQ2, is almost absent in the photoanodes sensitized with the 8:2 and 6:4 dye mixtures. With the decreasing molar ratio of SQ2 in the mixture of the dyes, the peak intensity also reduces around the red/NIR region. On the contrary, the peak intensity around 400 nm is not differing much with the varying molar ratios of the two dyes. The relative ratio of the dye loading is not exactly proportional to the ratio of the liquid cocktail in which the photoanodes were immersed. The most fascinating feature of this two particular dyes is that PPIX is serving the purpose of a co-adsorbent and efficiently preventing the aggregation of SQ2, which has been proven further by measuring the device performances.

**Figure 4:**
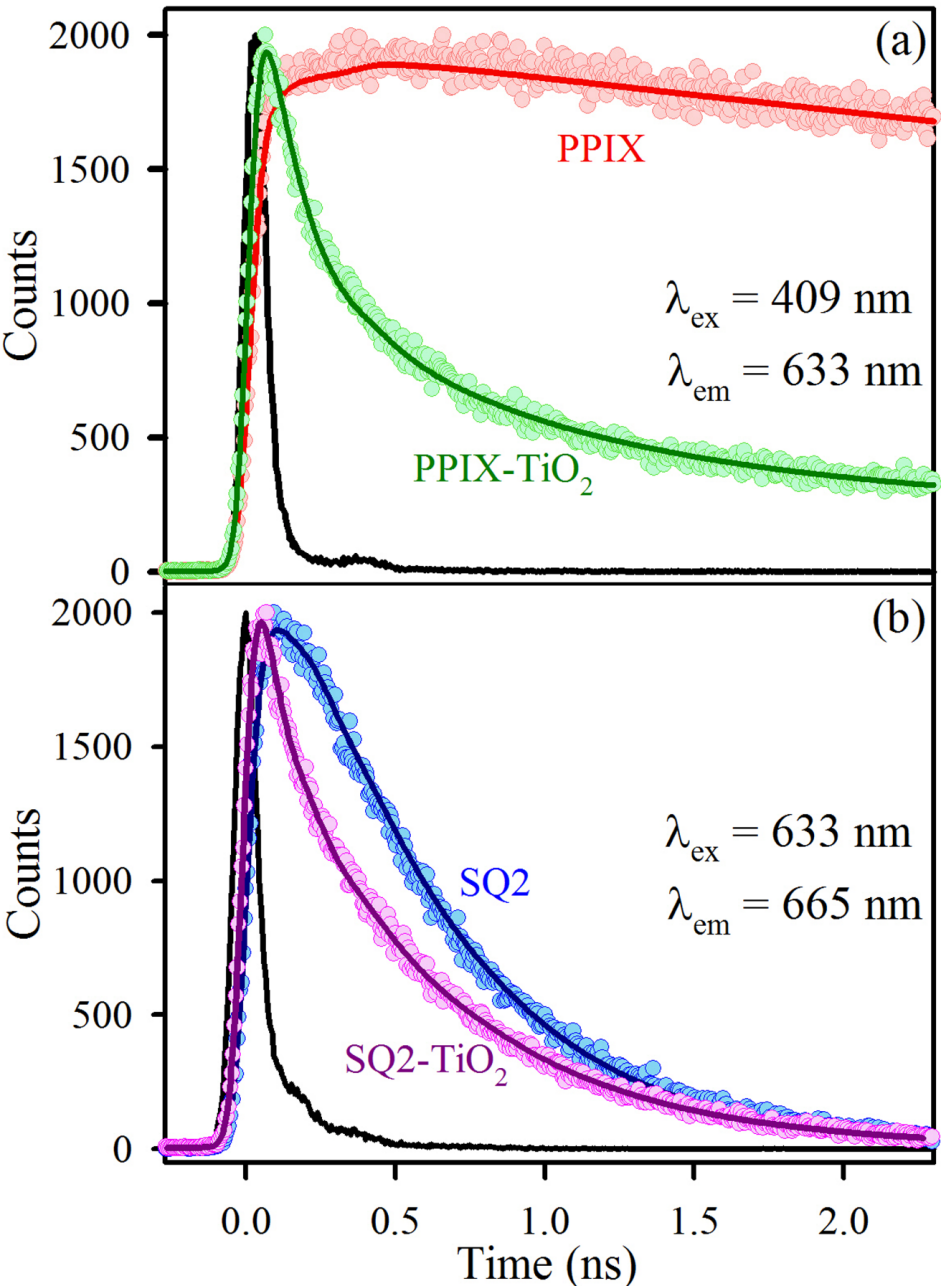
(a) Fluorescence decay profiles of PPIX (red) and PPIX attached to TiO_2_ (green), (b) fluorescence decay patterns of SQ2 (blue) and SQ2 attached to TiO_2_ (pink).

The electron injection efficiency from the dye to the semiconductor is one of the key factors that affect the efficiency of DSSCs. As shown in Figure 4a, time-resolved fluorescence transients were measured for a PPIX solution and a PPIX–TiO_2_ solution using DMSO as a solvent. A 409 nm laser was used as an excitation source and the emission decay was recorded at 633 nm. The decay of PPIX was single-exponential with a time scale of 14 ns. For PPIX–TiO_2_, a faster component of 130 ps (63%) was obtained, which is ascribed to be the electron transfer time scale from the excited state of PPIX to the conduction band of TiO_2_. Figure 4b shows that the decay of the excited state of SQ2 in ethanol was also single-exponential (500 ps) and a very fast electron transfer timescale of 60 ps (64%) was obtained after attachment of SQ2 on the TiO_2_ surface. All the lifetime components and their relative percentages are summarized in Table 2.

**Table 2:** Dynamics of picosecond-resolved fluorescence transients of the dyes PPIX, SQ2 and the nanohybrids.

sample	excitation wavelength (nm)	emission wavelength (nm)	τ_1_ (ns)	τ_2_ (ns)	τ_3_ (ns)

PPIX	409	633	14.00 (100%)	—	—
PPIX-TiO_2_	409	633	0.13 (63%)	0.89 (26%)	14.00 (11%)
SQ2	633	665	0.50 (100%)	—	—
SQ2-TiO_2_	633	665	0.06 (64%)	0.58 (36%)	—

**Figure 5:**
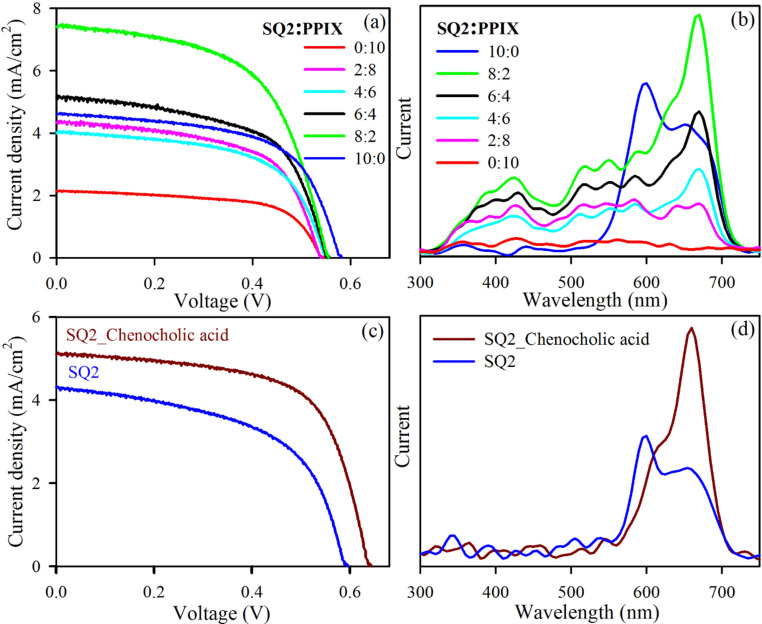
(a) *I*–*V* characteristics and (b) wavelength-dependent photocurrent of DSSCs sensitized with different ratios of SQ2 and PPIX. (c) *I*–*V* characteristics and (d) wavelength-dependent photocurrent for SQ2 and a mixture of SQ2 and 5 mM chenodeoxycholic acid.

Figure 5a presents the current density (*J*) vs voltage (*V*) characteristics of the co-sensitized DSSCs. Sample S8P2 shows the highest efficiency (2.4%) with a much higher photo-current than the cells sensitized by only one of the dyes. From Table 3, it can be noticed that we could reach an open-circuit voltage (*V*_oc_) of 0.58 V and an efficiency of 1.62% for the DSSC sensitized with only SQ2 (S10P0). For the DSSC sensitized with only PPIX (S0P10) the efficiency was 0.73% and *V*_oc_ was 0.55V. The *V*_oc_ values of the co-sensitized DSSCs were increasing from 0.55 to 0.58 V with increasing molar ratio of SQ2 as both dyes contribute to the Fermi level of the photoanode. The current density was observed to increase with increasing relative concentration of SQ2. For molar ratio of SQ2/PPIX of 6:4 and 8:2, the DSSCs exhibit higher photo currents and higher efficiencies than the one sensitized with only SQ2. The highest efficiency (2.4%) was observed for S8P2 with a current density of 7.4 mA/cm^2^. Besides the increase in absorption window, the FRET from PPIX to SQ2 is responsible for the significant increase in photocurrent at that particular ratio of the two dyes. This is evident from the photocurrent-vs-wavelength data of the DSSCs shown in Figure 5b. Even though the dye absorption in the visible region was shown to be almost the same for S8P2, S6P4 and S0P10 (Figure 3b), the measured photocurrent was prominently higher in the visible range for S8P2. It can be concluded that the optimum acceptor-to-donor ratio is 8:2 (SQ2/PPIX) and the excited-state energy of PPIX is transferred to SQ2 followed by the injection from the excited state of SQ2 to the conduction band of TiO_2_. Thus, FRET causes increased light harvesting in the visible region. Another notable feature in Figure 5b is the better harvesting in the NIR/red region of S8P2 although there is a lower concentration of SQ2 in S8P2 than in S10P0 (Figure 3b). The reason behind this anomaly is the much better light harvesting ability of the monomer of SQ2 compared to the aggregated dye. The photocurrent around 600 nm is harvested mainly by SQ2 dimers, but the aggregation promotes a lack of directionality of excited-state electrons of the dye. As a consequence the harvesting ability of the monomer around 650 nm reduces significantly. This fact is clearly manifested in the wavelength-dependent photocurrent measurement of S10P0 where a relatively higher photocurrent was observed at the absorption peak wavelength of the dimer of SQ2 than at that of the monomer. A noteworthy higher photon-to-current conversion in the absorption region of SQ2 was observed for S8P2 as PPIX reduces the aggregation of SQ2. The harvesting in the absorption region of the SQ2 monomer is observed to be remarkably higher than that in the dimer absorption region in the S8P2-sensitized DSSC, which reduces the possibility of self-quenching and consequently increases the overall photocurrent output. In earlier reports about SQ2-co-sensitized DSSCs chenodeoxycholic acid (CDCA) was proposed as a co-adsorbent to diminish SQ2 aggregation [32–33,41]. A detailed characterization of a SQ2–CDCA-sensitized DSSC was also carried out and the results are shown in Figure 5c,d. From the *I*–*V* values of the SQ2–CDCA-sensitized DSSC in Table 4, it can be noted that the efficiency was much lower than that of the S8P2-sensitized DSSC. Comparing the ratio of the photocurrent at the dimer and monomer absorption region of SQ2 in Figure 5b,d it can be concluded that S8P2 is the optimal composition. The value of *V*_oc_ of the CDCA–SQ2-sensitized DSSC was higher than that of S8P2, because CDCA does not contribute to the Fermi level unlike PPIX. The value of *J*_sc_ was strikingly higher in S8P2 than in the CDCA–SQ2-sensitized DSSC because of FRET from PPIX to SQ2. Thus, apart from getting a FRET-induced higher photocurrent, the idea of using one of the co-sensitizers as a co-adsorbent of the other is interesting as it is reducing the effective cost of the device.

**Table 3:** Solar cell performances using dyes with different molar ratios of SQ2 and PPIX as sensitizer.

sample SQ2/PPIX	FF (%)	*V*_oc_ (V)	*J*_sc_ (mA/cm^2^)	η (%)

0:10	63	0.55	2.1	0.73
2:8	60	0.54	4.3	1.39
4:6	64	0.55	4.0	1.31
6:4	60	0.56	5.7	1.66
8:2	57	0.57	7.4	2.40
10:0	61	0.58	4.6	1.62

**Table 4:** Performances of SQ2 sensitized and a mixture of SQ2 and 5 mM chenodeoxycholic acid sensitized solar cells.

sample	FF (%)	*V*_oc_ (V)	*J*_sc_ (mA/cm^2^)	η (%)

SQ2	54	0.60	4.3	1.38
SQ2–chenocholic acid (5 mM)	64	0.64	5.1	2.06

**Figure 6:**
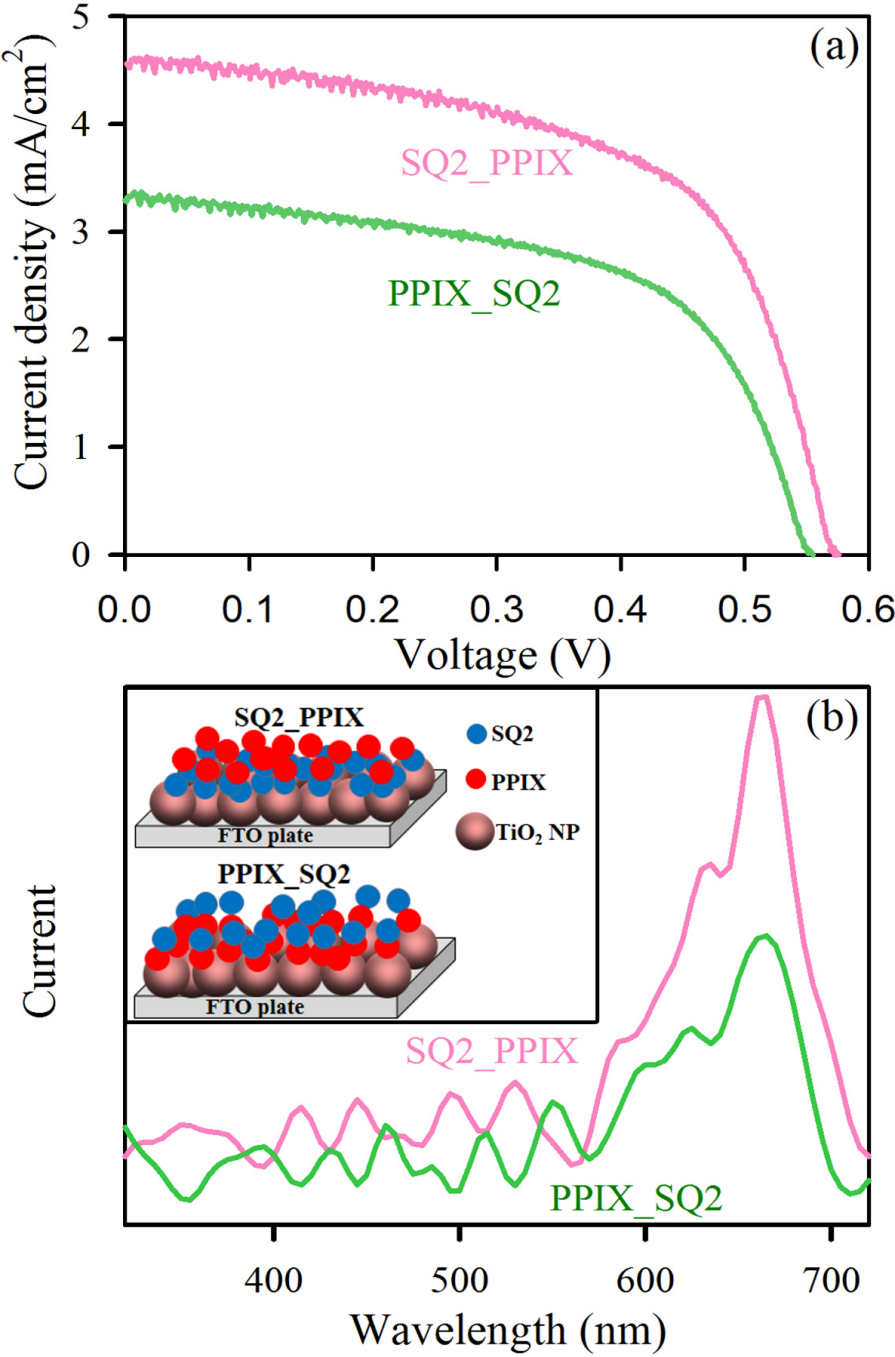
(a) *I*–*V* characteristics and (b) wavelength-dependent photocurrent response of the DSSCs with one-by-one dye sensitization. The inset shows the scheme of the sensitized photoanodes.

The enhancement in photocurrent due to dipole–dipole coupling is further verified by the sequential one-by-one sensitization of the two dyes where the first layer of the dye is supposed to be acting as the main electron-injecting sensitizer and the second dye layer will prefer to form some π-stacking attachment with the first dye layer as it would not get much vacancy on the TiO_2_ surface to be attached with directly. As shown in Figure 6a, SQ2_PPIX is showing higher efficiency and higher photocurrent than PPIX_SQ2. The efficiency and other solar cell parameters are summarized in Table 5. In SQ2_PPIX the first active layer of sensitizer is SQ2. So, PPIX can absorb the visible light and transfer the energy to SQ2, followed by electron injection from SQ2 to TiO_2_ in SQ2_PPIX which lead us to get a increased photocurrent. The process of FRET is not much facile in PPIX_SQ2 as the first active layer of sensitizer is the donor itself, in that case. It can also be demonstrated from the wavelength dependent photocurrent measurement of SQ2_PPIX and PPIX_SQ2 that the ratio of photocurrent in the absorption regions of the monomer and dimer of SQ2 is not comparable to that of S8P2. PPIX can efficiently perform as a co-adsorbent when it is in the cocktail mixture with the SQ2 unlike the one by one sensitization, as it could prevent the aggregation during the attachment of the sensitizers with TiO_2_.

**Table 5:** Performances of DSSCs using one-by-one sensitization of SQ2 and PPIX.

sample	FF (%)	*V*_oc_ (V)	*J*_sc_ (mA/cm^2^)	η (%)

SQ2 (12 h)–PPIX (12 h)	59	0.58	4.6	1.58
PPIX (12 h)–SQ2 (12 h)	59	0.55	3.3	1.09

## Conclusion

Detailed studies of the dynamics between the organic dyes PPIX and SQ2 have been performed using ultrafast spectroscopy, and FRET was observed from the donor PPIX to the acceptor SQ2. DSSCs have been fabricated by co-sensitizing the photoanodes with mixtures of the two dyes to achieve a reasonably good combined absorption solar light by these two dyes. The *I*–*V* measurement data exhibit a higher efficiency for the co-sensitized DSSCs than for the single-dye-sensitized DSSCs. A significantly enhanced photocurrent was obtained from the co-sensitized DSSCs at a particular ratio of the two sensitizers due to efficient dipole–dipole coupling. From the wavelength-dependent photocurrent measurements it has been observed that PPIX is successfully playing the role of an anti-aggregating agent of SQ2. It, consequently, increasing the light harvesting in the red/NIR region compared to the DSSC sensitized with only SQ2. The use of any additional co-adsorbent is not needed in the proposed co-sensitization, because PPIX is behaving simultaneously as a co-sensitizer and co-adsorbent. Thus, it can be asserted that PPIX has an impressive potential to increase the efficiency of DDSCs being a co-sensitizer with the NIR-absorbing SQ2 dye. Because the three essential requirements of efficient light harvesting, i.e., FRET, increased absorption window and suppression of aggregation, are concurrently fulfilled by PPIX.

## References

[1] Mathew S, Yella A, Gao P, Humphry-Baker R, Curchod B F E, Ashari-Astani N, Tavernelli I, Rothlisberger U, Nazeeruddin M K, Grätzel M (2014). Nat Chem.

[2] Graetzel M, Janssen R A J, Mitzi D B, Sargent E H (2012). Nature.

[3] Grätzel M (2009). Acc Chem Res.

[4] O’Regan B, Grfitzeli M (1991). Nature.

[5] Wang H, Wang B, Yu J, Hu Y, Xia C, Zhang J, Liu R (2015). Sci Rep.

[6] Zhang X, Wang S-T, Wang Z-S (2011). Appl Phys Lett.

[7] Duan Y, Fu N, Liu Q, Fang Y, Zhou X, Zhang J, Lin Y (2012). J Phys Chem C.

[8] Mali S S, Betty C A, Bhosale P N, Patil P S, Hong C K (2014). Sci Rep.

[9] Shiu J-W, Chang Y-C, Chan C-Y, Wu H-P, Hsu H-Y, Wang C-L, Lin C-Y, Diau E W-G (2015). J Mater Chem A.

[10] Tsao H N, Yi C, Moehl T, Yum J-H, Zakeeruddin S M, Nazeeruddin M K, Grätzel M (2011). ChemSusChem.

[11] Gonçalves L M, de Zea Bermudez V, Ribeiro H A, Mendes A M (2008). Energy Environ Sci.

[12] Connell A, Holliman P J, Davies M L, Gwenin C D, Weiss S, Pitak M B, Horton P N, Coles S J, Cooke G (2014). J Mater Chem A.

[13] Lin R Y-Y, Yen Y-S, Cheng Y-T, Lee C-P, Hsu Y-C, Chou H-H, Hsu C-Y, Chen Y-C, Lin J T, Ho K-C (2012). Org Lett.

[14] Stathatos E, Kosyachenko L A (2011). Dye Sensitized Solar Cells as an Alternative Approach to the Conventional Photovoltaic Technology Based on Silicon - Recent Developments in the Field and Large Scale Applications. Solar cells - dye-sensitized devices.

[15] Holliman P J, Mohsen M, Connell A, Davies M L, Al-Salihi K, Pitak M B, Tizzard G J, Coles S J, Harrington R W, Clegg W (2012). J Mater Chem.

[16] Han L, Islam A, Chen H, Malapaka C, Chiranjeevi B, Zhang S, Yang X, Yanagida M (2012). Energy Environ Sci.

[17] Kimura M, Nomoto H, Masaki N, Mori S (2012). Angew Chem, Int Ed.

[18] Sharma G D, Panda M K, Roy M S, Mikroyannidis J A, Gad E, Coutsolelos A G (2013). J Renewable Sustainable Energy.

[19] Lee C H, Kim S A, Jung M R, Ahn K-S, Han Y S, Kim J H (2014). Jpn J Appl Phys.

[20] Holliman P J, Davies M L, Connell A, Velasco B V, Watson T M (2010). Chem Commun.

[21] Xue Z, Wang L, Liu B (2013). Nanoscale.

[22] Hardin B E, Sellinger A, Moehl T, Humphry-Baker R, Moser J-E, Wang P, Zakeeruddin S M, Grätzel M, McGehee M D (2011). J Am Chem Soc.

[23] Basham J I, Mor G K, Grimes C A (2010). ACS Nano.

[24] Choi H, Santra P K, Kamat P V (2012). ACS Nano.

[25] Sarkar S, Makhal A, Lakshman K, Bora T, Dutta J, Kumar Pal S (2012). J Phys Chem C.

[26] Das S, Thanulingam T L, Thomas K G, Kamat P V, George M V (1993). J Phys Chem.

[27] Das S, Thomas K G, Thomas K J, Madhavan V, Liu D, Kamat P V, George M V (1996). J Phys Chem.

[28] Yum J-H, Walter P, Huber S, Rentsch D, Geiger T, Nüesch F, De Angelis F, Grätzel M, Nazeeruddin M K (2007). J Am Chem Soc.

[29] Li J, Wu W, Yang J, Tang J, Long Y, Hua J (2011). Sci China: Chem.

[30] Sharma G D, Kurchania R, Ball R J, Roy M S, Mikroyannidis J A (2012). Int J Photoenergy.

[31] Kar P, Maji T K, Sarkar P K, Sardar S, Pal S K (2016). RSC Adv.

[32] Lin L-Y, Yeh M-H, Lee C-P, Chang J, Baheti A, Vittal R, Thomas K J, Ho K-C (2014). J Power Sources.

[33] Chang J, Lee C-P, Kumar D, Chen P-W, Lin L-Y, Thomas K J, Ho K-C (2013). J Power Sources.

[34] Sardar S, Sarkar S, Myint M T Z, Al-Harthi S, Dutta J, Pal S K (2013). Phys Chem Chem Phys.

[35] Sardar S, Kar P, Pal S K (2014). J Mater NanoSci.

[36] Lakowicz J R (1999). Principles of Fluorescence Spectroscopy.

[37] Sardar S, Kar P, Remita H, Liu B, Lemmens P, Pal S K, Ghosh S (2015). Sci Rep.

[38] Sardar S, Kar P, Sarkar S, Lemmens P, Pal S K (2015). Sol Energy Mater Sol Cells.

[39] Sarkar P K, Polley N, Chakrabarti S, Lemmens P, Pal S K (2016). ACS Sens.

[40] Leach S J, Scheraga H A (1960). J Am Chem Soc.

[41] Ren X, Feng Q, Zhou G, Huang C-H, Wang Z-S (2010). J Phys Chem C.

